# Supplementary material for: A Critical View of the Application of the APEX Software (Aqueous Photochemistry of Environmentally-Occurring Xenobiotics) to Predict Photoreaction Kinetics in Surface Freshwaters
Source: Molecules. 2019 Dec 18;25(1):9. doi: 10.3390/molecules25010009 (PMC7017383; doi:10.3390/molecules25010009)
Supplement: Supplementary file 1 [file molecules-25-00009-s001.zip › 3.2.4_gcc-4.4.0.zip!/3.2.4_gcc-4.4.0/3.2.4_gcc-4.4.0/doc/octave/html/interpreter/Statistical-Plots.html]

Statistical Plots - Untitled


Next: Tests,
Previous: Basic Statistical Functions,
Up: Statistics


---

### 25.3 Statistical Plots


Octave can create Quantile Plots (QQ-Plots), and Probability Plots
(PP-Plots). These are simple graphical tests for determining if a
data set comes from a certain distribution.

Note that Octave can also show histograms of data
using the `hist` function as described in
Two-Dimensional Plots.

— Function File: [q, s] = **qqplot** (x, dist, params)  
> Perform a QQ-plot (quantile plot).
>
> If F is the CDF of the distribution dist with parameters
> params and G its inverse, and x a sample vector of length
> n, the QQ-plot graphs ordinate s(i) = i-th
> largest element of x versus abscissa q(if) = G((i -
> 0.5)/n).
>
> If the sample comes from F except for a transformation of location
> and scale, the pairs will approximately follow a straight line.
>
> The default for dist is the standard normal distribution. The
> optional argument params contains a list of parameters of
> dist. For example, for a quantile plot of the uniform
> distribution on [2,4] and x, use
>
> ```
>           qqplot (x, "uniform", 2, 4)
> ```
>
> dist can be any string for which a function dist\_inv
> that calculates the inverse CDF of distribution dist exists.
>
> If no output arguments are given, the data are plotted directly.

— Function File: [p, y] = **ppplot** (x, dist, params)  
> Perform a PP-plot (probability plot).
>
> If F is the CDF of the distribution dist with parameters
> params and x a sample vector of length n, the
> PP-plot graphs ordinate y(i) = F (i-th largest
> element of x) versus abscissa p(i) = (i -
> 0.5)/n. If the sample comes from F, the pairs will
> approximately follow a straight line.
>
> The default for dist is the standard normal distribution. The
> optional argument params contains a list of parameters of
> dist. For example, for a probability plot of the uniform
> distribution on [2,4] and x, use
>
> ```
>           ppplot (x, "uniform", 2, 4)
> ```
>
> dist can be any string for which a function dist\_cdf
> that calculates the CDF of distribution dist exists.
>
> If no output arguments are given, the data are plotted directly.
